# The Calpain/Calpastatin System Has Opposing Roles in Growth and Metastatic Dissemination of Melanoma

**DOI:** 10.1371/journal.pone.0060469

**Published:** 2013-04-02

**Authors:** Quentin Raimbourg, Joëlle Perez, Sophie Vandermeersch, Aurélie Prignon, Guillaume Hanouna, Jean-Philippe Haymann, Laurent Baud, Emmanuel Letavernier

**Affiliations:** 1 Unité Mixte de Recherche 702 (UMR S 702), Université Pierre-et-Marie-Curie Paris VI et Institut National de la Santé et de la Recherche Médicale, Hôpital Tenon, Paris, France; 2 Département de Médecine Nucléaire et Université Pierre-et-Marie-Curie Paris VI, Hôpital Tenon, Paris, France; 3 Unité Mixte de Recherche 702 (UMR S 702), Université Pierre-et-Marie-Curie Paris VI, Institut National de la Santé et de la Recherche Médicale et Assistance Publique des Hôpitaux de Paris, Hôpital Tenon, Paris, France; Istituto Superiore di Sanità, Italy

## Abstract

Conventional calpains are ubiquitous cysteine proteases whose activity is promoted by calcium signaling and specifically limited by calpastatin. Calpain expression has been shown to be increased in human malignant cells, but the contribution of the calpain/calpastatin system in tumorigenesis remains unclear. It may play an important role in tumor cells themselves (cell growth, migration, and a contrario cell death) and/or in tumor niche (tissue infiltration by immune cells, neo-angiogenesis). In this study, we have used a mouse model of melanoma as a tool to gain further understanding of the role of calpains in tumor progression. To determine the respective importance of each target, we overexpressed calpastatin in tumor and/or host in isolation. Our data demonstrate that calpain inhibition in both tumor and host blunts tumor growth, while paradoxically increasing metastatic dissemination to regional lymph nodes. Specifically, calpain inhibition in melanoma cells limits tumor growth in vitro and in vivo but increases dissemination by amplifying cell resistance to apoptosis and accelerating migration process. Meanwhile, calpain inhibition restricted to host cells blunts tumor infiltration by immune cells and angiogenesis required for antitumor immunity, allowing tumor cells to escape tumor niche and disseminate. The development of highly specific calpain inhibitors with potential medical applications in cancer should take into account the opposing roles of the calpain/calpastatin system in initial tumor growth and subsequent metastatic dissemination.

## Introduction

Calpains are ubiquitous cysteine proteases activated by calcium signaling and/or epidermal growth factor (EGF) and vascular endothelial growth factor (VEGF) [1]–[3]. The term “calpain” usually refers to “conventional” or “typical” µ- and m-calpains, which heterodimerize with calpain 4 or calpain small subunit 1 (Css1), their regulatory subunit [1]. Other calpain isoform expression is limited and tissue-specific. Both m- and µ-calpain play essential roles in cellular homeostasis. For instance, they promote cell motility by modulating cytoskeleton organization through limited proteolysis, increase inflammation by facilitating NF-κB activation and leukocyte diapedesis, or promote angiogenesis through various mechanisms [4]–[9]. A dramatic activation of calpain results in cell death by apoptosis and necrosis [10], [11]. Calpain intracellular activity is however limited by their specific and ubiquitous inhibitor calpastatin [1]. Although calpain does not conform to a consensus cleavage site but recognizes a broad spectrum of PEST sequences, µ- and m-isoforms share a similar catalytic site and have redundant substrates in cells. Actually, targeted disruption of *Capn1* gene encoding µ-calpain does not result in any phenotype in mice, with the exception of platelet dysfunction, whereas targeted disruption of *Capns1* gene encoding Css1 is lethal [12], [13]. By contrast, mice knock out (KO) for *Capn2* gene encoding m-calpain are not viable from an embryonic stage but the underlying mechanisms remain mysterious [14].

The contribution of the calpain/calpastatin system in tumorigenesis remains unclear [15]. First, conventional calpain expression has been shown to be increased in human malignant cells, such as breast cancer, schwannoma, meningioma or colorectal cancer [16]–[18]. Expression levels of µ-calpain and Css1 correlate with renal cell carcinoma malignancy and hepatocarcinoma invasiveness, respectively [19], [20]. In parallel, the calpastatin expression is increased in endometrial cancer [21]. Tissue-specific calpain isoforms may also be implied in some tumor development. Calpain 9 expression is decreased in gastric cancer, deletion of *CAPN9* gene encoding calpain 9 promotes the onset of malignant fibroblasts in culture, and *CAPN10* gene is involved in digestive and laryngeal cancers [22]–[24]. Interestingly, splicing variants of calpain 3, the muscle-specific isoform, are expressed in melanoma cells and their expression is downregulated in highly aggressive lesions [25].

Calpains may exert opposing roles in tumor progression. On the one hand, calpain activation could promote oncogenesis. Calpains degrade tumor suppressors such as p53 or the *NF2* gene product Merlin [17], [26]. Both µ- and m-calpains cleave IκBα in a specific PEST sequence, allowing NF-κB translocation into nucleus, thus promoting cell survival [7]. Calpains are also essential for cytoskeleton reorganization and therefore promote cell migration and invasiveness, which are essential features of tumor cells [27]. Moreover, calpains are essential for VEGF-response and angiogenesis and could therefore promote tumor vascularization [3], [8], [9]. On the other hand, calpains may also exert protective effects against cancer. Calpains interfere with the Wnt/β-catenin pathway by degrading β-catenin in the cytoplasm, thus limiting the transcription of genes involved in cancer [28]. Calpains activate caspases, particularly caspase 7, inducing cell apoptosis [29]. They also cleave Bcl-2, leading to BAX translocation into mitochondria, or degrade c-fos, c-jun, cain/cabin 1 what results in cell death [30]–[32]. Calpains perform a limited proteolysis of apoptosis-inducing factor (AIF), promoting its mitochondrial release and resulting in apoptosis/necroptosis [33]. In addition, calpains could be an important link between apoptosis and (macro-)autophagy, a process removing damaged proteins and organelles from cells. Autophagy acts either as a promoter or an inhibitor of tumorigenesis depending on tumor type or stage, suggesting that calpains could be either pro- or anti-tumoral according to the context [34]. At last, we have previously shown that µ- and m-calpain are highly expressed in T-cells and play an important role in allograft rejection [35]. Since immunity is essential to control malignant tumors, it may be hypothesized that calpains are involved in immunologic response against tumor cells.

Thus, in this study, we have used a mouse model of melanoma as a tool to gain further understanding of the role of calpains in tumor progression. Since specificity of available calpain inhibitors remains questionable, we compared melanoma progression in wild type (WT) mice and transgenic mice overexpressing calpastatin (CalpTG), which specifically inhibits both conventional isoforms [9], [35]–[38]. Calpains are critically involved in tumor promotion and progression by targeting tumor cells themselves (oncogenesis, growth, migration, death) and/or tumor niche (immune response, proangiogenic events). To determine the respective importance of each target, we then overexpressed calpastatin in tumor or host in isolation, by using a melanoma cell line from the same C57BL/6 genetic background than tumor bearing mice. Our data demonstrate that calpain inhibition actually increases melanoma dissemination, mainly by decreasing apoptosis and increasing mobility of melanoma cells while limiting markedly infiltration of immune cells in tumor niche.

## Results

### Calpastatin transgene expression limits melanoma growth

**Figure 1:**
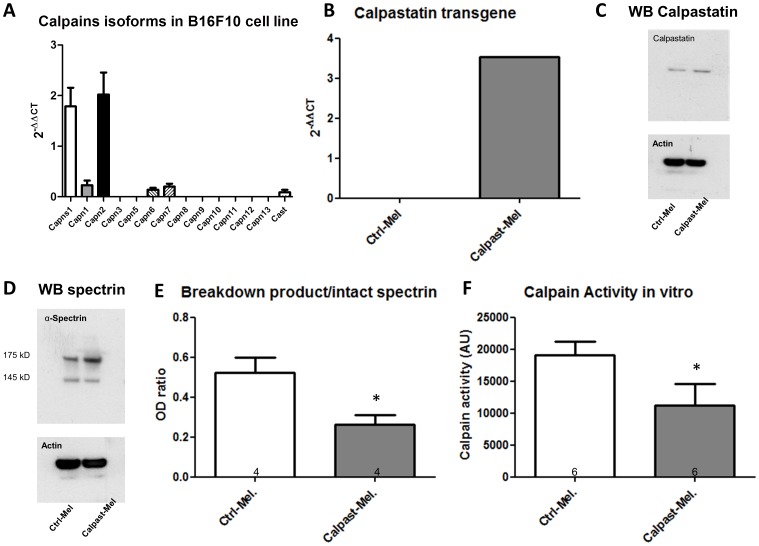
Calpain isoforms and calpastatin transgenic expression in B16-F10 cell line in culture. **A.** Calpain isoform transcripts were quantified by PCR. The most abundant was m calpain (*Cpn2*) followed by µ calpain transcript (*Cpn1*) and their common sub-unit calpain 4 transcript (*Cpns1*). Calpastatin transcript (*Cast1*) was also detected. Means and error bars correspond to 4 independent RNA extracts. *Gapdh* was the housekeeping gene used for normalization. **B.** Calpastatin rabbit transgene expression was assessed by quantitative PCR. Calpastatin transgene was stably and efficiently expressed in B16-F10 clone (Calpast-Mel) but not in control B16-F10 clones (Ctrl-Mel). **C.** Calpastatin expression was assessed by Western Blot. Calpastatin protein was overexpressed in Calpast-Mel clones. **D and E.** Spectrin intact form and calpain-dependent breakdown products were assessed by Western Blot and optical density measure (OD). Calpastatin overexpression (Calpast-Mel) decreased spectrin degradation by calpains. N = 4 independent experiments, * p<0.05. **F**. Calpain activity in vitro was measured by specific substrate fluorescence. Calpain activity was decreased by calpastatin transgene. N = 6 independent experiments, * p<0.05.

To determine the role of calpains in melanoma progression, we subcutaneously implanted WT and CalpTG mice with the highly metastatic mouse melanoma cell line B16-F10. We took advantage that this cell line shares the same C57BL/6 background than WT and CalpTG mice, avoiding bias due to allograft immune reactivity. Calpain expression and activity in WT and CalpTG mice have been previously characterized [36]. Here, we characterized calpain isoforms transcripts in vitro in B16-F10 cells by quantitative polymerase chain reaction (qPCR). *Capn 6* and *7* isoforms transcripts were detected at significant levels but not *Capn 3, 8, 9, 10, 11, 12* and *13* transcripts. *Capn1, Capn2* and C*apns* transcripts, coding for calpains µ, m and the common regulatory sub-unit Css1 respectively, were abundant, especially *Capn2* transcript. *Cast* gene transcript coding for calpastatin was also expressed in B16-F10 (Figure 1A). Overall, the respective levels of calpains transcripts in B16-F10 cells were similar to those observed in mice tissues such as kidneys (not shown).Spontaneous calpain activity in B16-F10 cells was high when compared to non-malignant cell lines (not shown). In order to inhibit this activity, a stable transfection of the same calpastatin transgene previously used to create transgenic mice was performed. Transfected melanoma cells (Calpast-Mel) expressed the rabbit calpastatin transgene (Figures 1B and 1C). Compared to control cells transfected with an empty vector (Ctrl-Mel), calpain activity in these cells was significantly limited as assessed by measuring both in vitro the cleavage of a fluorescent substrate and in vivo the accumulation of α-spectrin breakdown products (Figures 1D-F).

**Figure 2:**
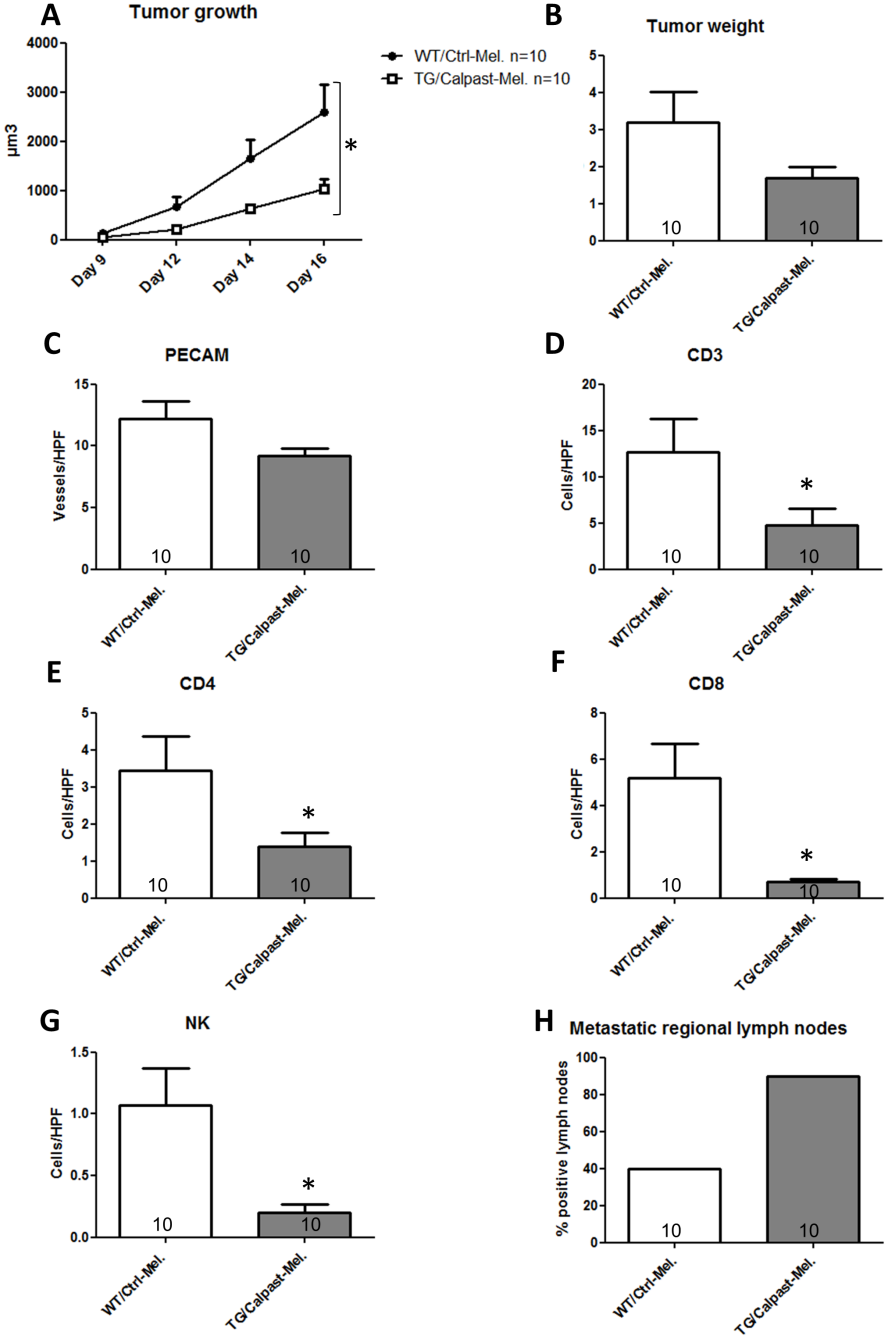
Inhibition of calpains in both host and melanoma cells (global inhibition). C57BL/6 control (WT mice) and transgenic mice (CalpTG mice) were injected with one million melanoma B16-F10 cells transfected with control plasmid (WT/Ctrl-Mel) and calpastatin plasmid (TG/Calpast-Mel) respectively. Mice were sacrificed at day 16 for tissue analysis. **A**. Tumor growth was measured from day 9 to day 16. Calpastatin overexpression in both hosts and melanoma decreased significantly tumor growth. N = 10/group, * p<0.05. **B**. Tumor weight at day 16. Calpastatin overexpression in both hosts and melanoma decreased non-significantly tumor weight. N = 10/group, p = NS. **C**. Angiogenesis assessed by vessel count at 200×magnification after CD-31 staining. TG/Calpast-Mel mice had a trend to have less neo-angiogenesis than WT mice. N = 10/group, p = NS. **D,E,F,G.** CD3, CD4, CD8 and NK cell number/HPF (200×magnification). Immune cell infiltrate was significantly lower in TG/calpast Mel mice than in WT/Ctrl mice. N = 10/group, * p<0.05. **H.** Proportion of metastatic regional lymph nodes at day 16. TG/Calpast-Mel mice had a trend to have more metastatic lymph nodes than WT mice (9/10 vs 4/10 respectively, p = NS).

In a first study, we compared 10 WT mice receiving control melanoma cells (WT/Ctrl-Mel) and 10 CalpTG mice injected with transgenic melanoma cells (TG/Calpast-Mel). Primary tumor size was analyzed from day 9 to day 16 after cell injection and their weight was measured on the day of the sacrifice. Tumor growth was significantly reduced in TG/Calpast-Mel as compared to WT/Ctrl-Mel (p<0.01, n = 10/group Figure 2A). At day 16, there was a trend toward a lower tumor weight in the TG/Calpast-Mel group as compared to the WT/Ctrl-Mel group (1.7±0.3 vs 3.2±0.8 g, n = 10/group, p = NS Figure 2B). There was a trend toward less vascular density in the TG/Calpast-Mel group (9.2±0.5 vs 12.2±1.4 vessels/HPF, p = 0.06, n = 10/group, Figure 2C). CD3 infiltrate was significantly reduced in TG/Calpast-Mel mice (p  = 0.03, n = 10/group, Figure 2D). In addition, CD4 and NK cell recruitment was significantly reduced in TG/Calpast-Mel animals (p  = 0.02 and p = 0.004, respectively, n = 10/group, Figures 2E and 2F) and the reduction of CD8 cells in this group was particularly marked (0.7±0.1 vs 5.2±1.5 cells/HPF, p = 0.0003, n = 10/group, Figure 2G). Pathological examination revealed no metastasis in organs including lungs or liver at that time. Metastatic regional (axillary) lymph nodes were observed in 90% of TG/Calpast-Mel mice but only 40% of WT/Ctrl-Mel mice (p = 0.057, n = 10/group, Figure 2H). Collectively, these data suggest that calpain inhibition by calpastatin overexpression limits melanoma growth, vascularization, and immune cell infiltrate but paradoxically amplifies its dissemination to regional lymph nodes. Such opposing effects could be explained by different roles of calpains in tumor cells and host cells.

### Calpastatin transgene expression restricted to melanoma cells limits primary tumor growth while increasing its dissemination to regional lymph nodes

**Figure 3:**
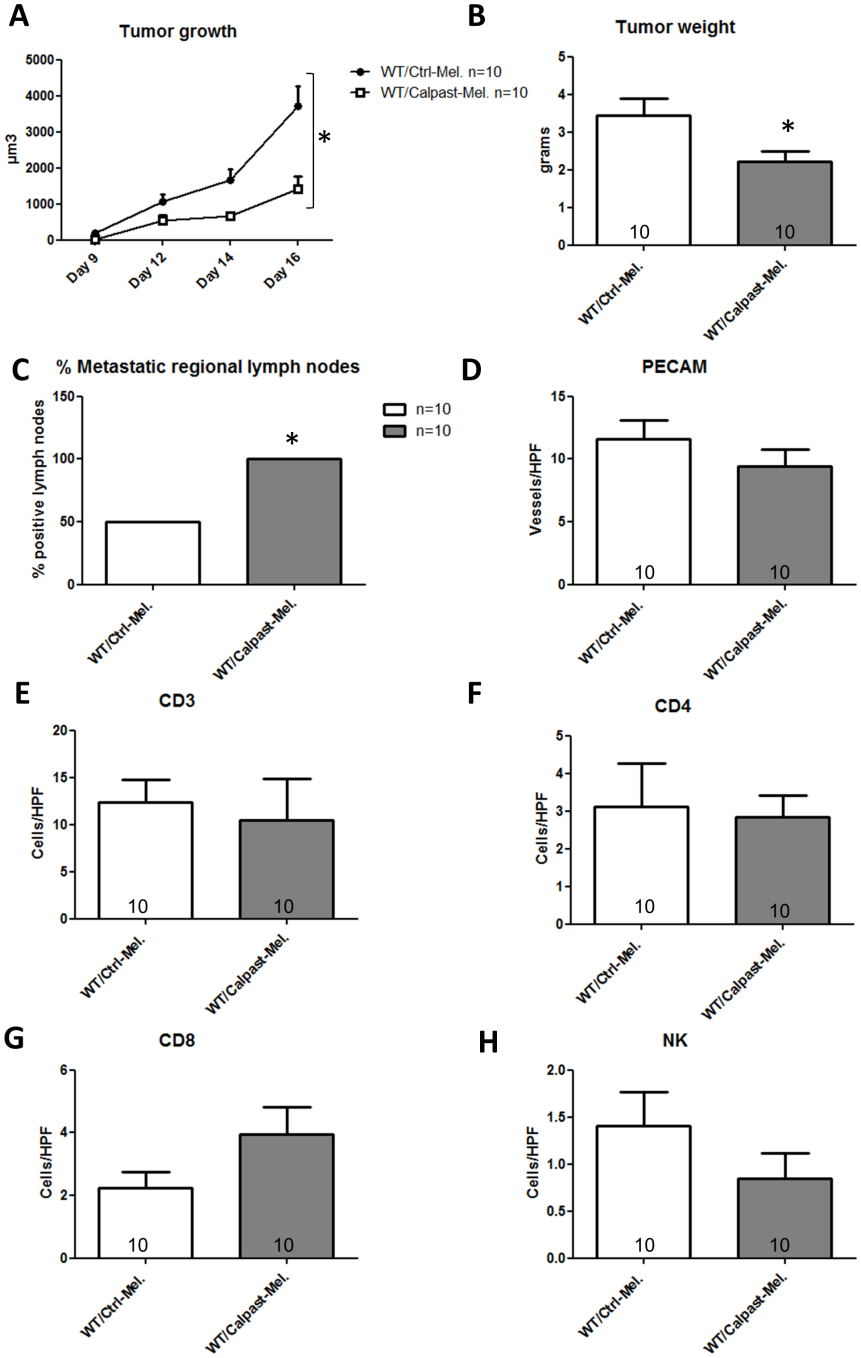
Inhibition of calpains in melanoma cells only: an in vivo approach. C57BL/6 WT mice were injected with one million melanoma B16-F10 cells either transgenic for calpastatin (Calpast-Mel) or transfected with a control plasmid (Ctrl-Mel) and sacrificed at day 16 for tissues analysis. **A**. Tumor growth was measured from day 9 to day 16. Calpastatin overexpression in melanoma decreased significantly tumor growth. N = 10/group, * p< 0.05. **B**. Tumor weight at day 16. Calpastatin overexpression reduced significantly melanoma weight. N = 10/group, * p<0.05. **C**. Proportion of metastatic regional (axillary) lymph nodes at day 16. Mice injected with melanoma cells with reduced calpain activity (Calpast-Mel) had significantly more metastatic lymph nodes than controls (Ctrl-Mel) (10/10 vs 5/10 respectively, * p<0.05). **D**. Angiogenesis assessed by vessel count at 200×magnification after CD-31 (PECAM) staining. Neo-angiogenesis was similar in transgenic and control melanomas. N = 10/group, p = NS. **E,F,G,H**: CD3, CD4, CD8 and NK cell number/HPF (200×magnification). Immune cell infiltrate was similar in transgenic and control tumors. N = 10/group, p = NS.

Given the specific roles of calpains in tumor and tumor microenvironment, we analyzed first the importance of calpains in melanoma cells by injecting Ctrl-Mel or Calpast-Mel to WT mice. Tumor growth was significantly slower in animals injected with Calpast-Mel cells as compared to animals receiving Ctrl-Mel cells (p<0.01, n = 10/group Figure 3A), as well as tumor weight at day 16 (2.22±0.28 vs 3.43±0.49 g, respectively, p = 0.03, n = 10/group, Figure 3B). All the WT mice receiving Calpast-Mel cells exhibited metastatic regional lymph nodes whereas only 5/10 animals receiving control melanoma had metastatic regional lymph nodes (p = 0.032, n = 10/group, Figure 3C). We did not find any difference between the 2 groups in tumor vascular density (Figure 3D, n = 10/group) and in the number of infiltrating immune cells, including CD3, CD4, CD8, and NK cells (Figure 3E-H, n = 10/group).

**Figure 4:**
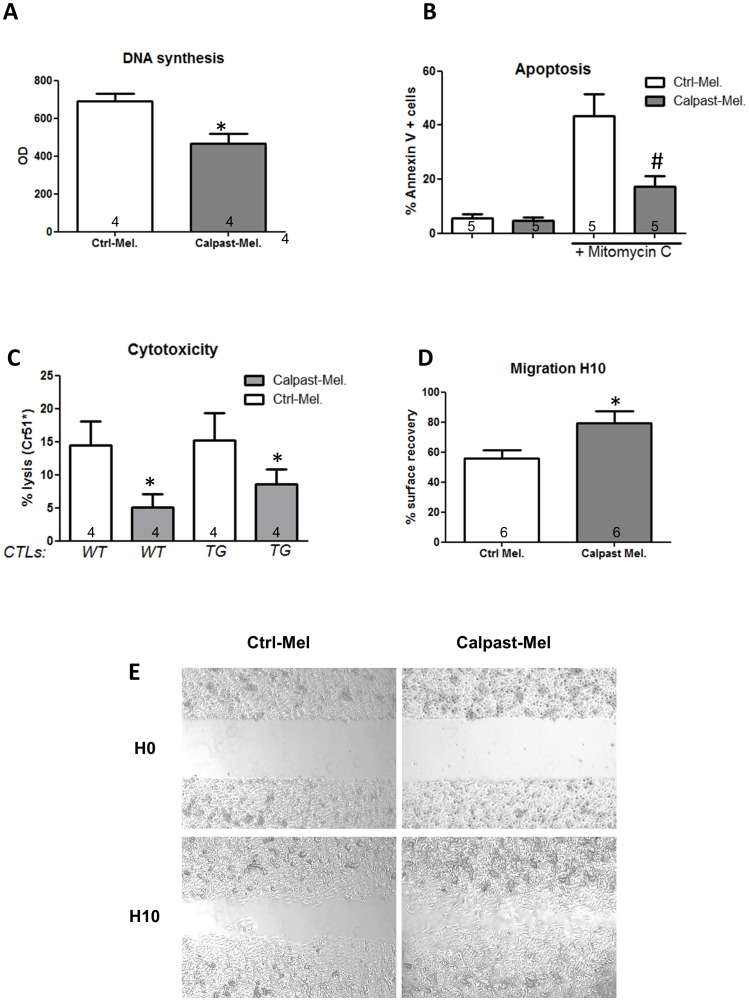
Inhibition of calpains in melanoma cells only: an in vitro approach. **A**. Cellular proliferation measured by BrdU incorporation (DNA synthesis). Calpastatin overexpression reduced significantly DNA synthesis under basal culture conditions. N = 4 independent experiments, * p<0.05. **B**. Quantification of apoptotic cells by Annexin V (+/- propidium iodide) staining by flow cytometry under basal conditions and after 24 hours exposure to mitomycin C. Melanoma cells overexpressing calpastatin were protected against mitomycin C-induced apoptosis. N = 5 independent experiments, # p<0.05 vs Ctrl-Mel+ Mitomycin C. **C**. Cytotoxic effect of previously immunized splenocytes (CTLs, previously immunized against control B16F10cells) from C57BL/6 mice (WT) or calpastatin transgenic mice (TG) against B16-F10 melanoma cells transfected with calpastatin (Calpast-Mel) or control plasmid (Ctrl-Mel). Cytotoxicity was measured by chromium release after incorporation in melanoma cells. TG CTLs and WT CTLs cells exerted a similar cytolytic response (p = NS) but Calpast-Mel cells were partly protected against immune effectors as compared to Ctrl-Mel cells. N = 4 independent experiments, * p<0.05. **D, E**. Melanoma cell migration measured by cell free gap surface recovery 10 hours after removing insert. Calpastatin overexpression increased significantly cellular migration properties in vitro. N = 6 independent experiments, * p<0.05.

To further define the mechanisms whereby the calpain/calpastatin system would modify tumor size and metastatic properties, we studied in vitro the influence of calpastatin transgene expression on cell proliferation, spontaneous or induced apoptosis, and migration. As assessed by measuring BrdU incorporation, proliferation of Calpast-Mel cells was decreased compared to Ctrl-Mel cells, consistent with our in vivo observation (p = 0.028, n = 4 experiments, Figure 4A). To assess whether calpastatin transgene in melanoma cells affects apoptosis, we measured propidium iodide-annexin V staining by flow cytometry. Apoptosis rate was very low in both cell lines in culture conditions. Apoptosis was therefore induced by a classical agent, mitomycin C. Interestingly, the decrease of calpain activity induced by calpastatin over-expression significantly reduced the apoptosis induced by mitomycin C (p = 0.032, n = 5 experiments, Figure 4B). To further examine the potential importance of this resistance to apoptosis in a more physiological model and to analyze the cytolytic potential of WT and CalpTG immune cells, we compared lymphocyte-mediated cytotoxicity against Calpast-Mel cells and Ctrl-Mel cells. To this aim, we immunized C57BL/6 WT and CalpTG mice against control B16F10 melanoma cells during 10 days. We next analyzed in vitro the cytolytic potential of their splenocytes against Calpast-Mel cells and Ctrl-Mel cells in a ^51^Cr-release assay. The cytotoxic effects of splenocytes from WT and CalpTG mic were similar but significantly less marked on Calpast-Mel cells compared to Ctrl-Mel cells (p = 0.018, n = 4 experiments, Figure 4C). Surprisingly, calpastatin transgene increased melanoma cell migration properties, as evaluated in monolayer repair assay (p = 0.026 at 10 h, n = 6 experiments, Figures 4D and 4E). These results demonstrate that limiting calpain activity in melanoma cells is sufficient to blunt their proliferation while increasing their resistance to apoptosis and their mobility, thus facilitating metastatic dissemination.

### Calpastatin transgene expression restricted to host cells increases mainly melanoma cell dissemination to regional lymph nodes

**Figure 5:**
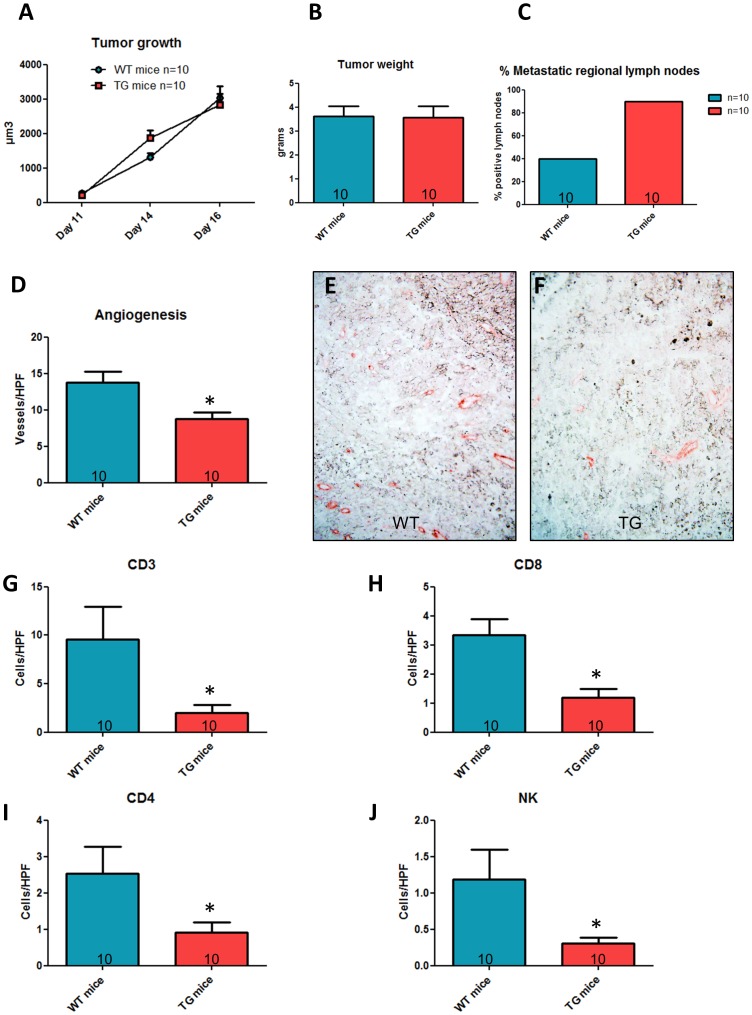
Inhibition of calpains in host mice only. C57BL/6 control (WT mice) or transgenic mice (CalpTG mice) were injected with one million melanoma B16F10 cells and sacrificed at day 16 for tissue analysis.**A**. Tumor growth was measured from day 11 to day 16. Calpastatin overexpression in host did not modify tumor growth. N = 10/group. **B**. Tumor weight at day 16. Calpastatin overexpression in hosts did not modify tumor weight. N = 10/group, p = NS. **C**. Proportion of metastatic regional lymph nodes at day 16. CalpTG mice had a trend to have more metastatic lymph nodes than WT mice (9/10 vs 4/10 respectively, N = 10/group, p = NS). **D,E,F**. Angiogenesis as assessed by vessel count at 200×magnification after CD-31 staining. Neo-angiogenesis was significantly decreased in CalpTG mice when compared to WT mice. N = 10/group, * p<0.05. **G,H,I,J**: CD3, CD4, CD8 and NK cell number/HPF (200×magnification). Immune cell infiltrate was significantly lower in CalpTG mice than in WT mice. N = 10/group, * p<0.05.

We and others have previously shown that calpain activity is reduced in tissues from CalpTG mice, including skin and immune cells [9], [35]–[38]. To investigate the functional consequence of calpastatin transgene expression in host cells, Ctrl-Mel cells were injected subcutaneously in either WT or CalpTG mice. There was no difference in tumor size or weight between CalpTG and WT mice (Weight: 3.56±0.50 and 3.61±0.46 g, respectively, Figures 5A and 5B, n = 10/group). Interestingly, CalpTG animals had a strong trend to have more metastatic regional lymph nodes compared to WT mice (90% vs 40%, p = 0.057, Figure 5C, n = 10/group). Neoangiogenesis was significantly decreased in CalpTG mice, the number of tumor vessels/HPF being 8.7±0.9 as compared to 13,7±1,7 in WT mice (p = 0,02, Figures 5D-F, n = 10/group). Immunostaining of tumors revealed significantly less tumor associated immune response in CalpTG animals as assessed by analyzing the number of CD3+, CD4+, CD8+ and NK cells (Figures 5G-J, n = 10/group). However, calpain inhibition in CALP TG immune cells did not modify their in vitro cytolytic potential against melanoma cells (Figure 4C, n = 4 experiments). Thus, the increase in metastatic regional lymph nodes in CalpTG mice may result mainly from a decrease of immune cell migration toward tumors, as we previously observed toward allograft in acute rejection process [35].

### Survival Studies

**Figure 6:**
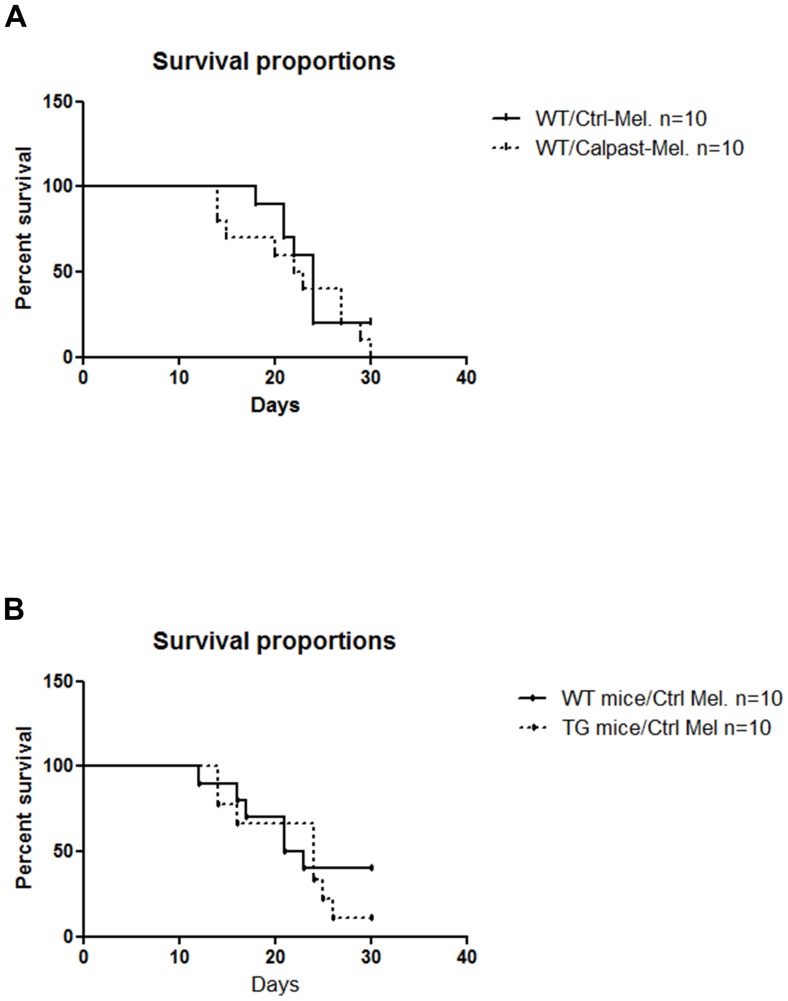
Survival studies. **A**. Specific limitation of calpain activity in melanoma cells only by calpastatin overexpression in vivo. C57BL/6 WT mice were injected with one million melanoma B16-F10 cells either transgenic for calpastatin (Calpast-Mel) or transfected with a control plasmid (Ctrl-Mel), n = 10/group. Limitation of calpain activity in melanoma cells only did not modify survival at day 30. **B**. Specific limitation of calpain activity in mice transgenic for calpastatin. C57BL/6 control (WT mice) or transgenic mice (CalpTG mice) were injected with one million control melanoma B16F10 cells, n = 10/group. Limitation of calpain activity in host did not modify survival at day 30.

Since calpain inhibition restricted to melanoma cells limits tumor growth but also increase melanoma cell resistance to apoptosis and dissemination toward lymph nodes, we analyzed the effect of calpastatin transgene expression in melanoma cells on mice survival. We did not observe any significant difference between the 2 groups (Figure 6A, n = 10/group). Since calpain inhibition restricted to host cells limits angiogenesis but also immune cell infiltrate, we determined whether calpastatin transgene expression in host cells would modify mice survival. Once more, we did not evidence any significant difference between the 2 groups (Figure 6B, n = 10/group).

## Discussion

The role of calpains in tumor induction and progression has been addressed before [15]–[18]. Main studies report that calpains increase while calpastatin limits tumor dissemination [19], [20], [39]. In contrast, our results have indicated that the specific inhibition of calpains activity by calpastatin accelerates metastatic dissemination to regional lymph nodes. Our results were obtained by analyzing the progression of melanoma in WT and CalpTG mice injected subcutaneously with the highly metastatic B16-F10 cell line which shares the same C57BL/6 genetic background with tumor bearing mice. The design of our studies allows (i) to specifically inhibit calpain activity using calpastatin and (ii) for the first time, to discriminate the roles of the calpain/calpastatin system in tumor cells (growth, death, migration) and in host (angiogenesis, immune response).

As stated above, we have chosen to inhibit both µ- and m-calpain isoforms by overexpressing calpastatin, since they share similar catalytic sites. Although our study was not designed to focus on µ- and m-calpain specificities, several levels of evidence argue for a dominant role of m-calpain in tumor cell physiology. First, we observed that m-calpain transcript expression was much higher than µ-calpain transcript one. Second, it has been shown that EGF-receptor signalling activates m-calpain rather than µ-calpain through MAP-kinase pathway [2]. It would be of interest to assess whether anti-oncogenic properties of EGF-R inhibitors are partly mediated by m-calpain repression. Third, m-calpain but not µ-calpain has been identified recently as a major actor of intrinsic or acquired resistance to chemotherapies in a colon cancer model, resulting in tumor growth through NF-κB activation [40]. At last, a recent study evidenced that m-calpain specific inhibition by Si-RNA in a mouse mammary carcinoma cell line was sufficient to reduce cellular proliferation [41]. The latter results are of interest since calpain involvement in cell cycle depends on cell line. We have previously observed that calpain inhibition decreases endothelial cell proliferation but does not impair kidney epithelial cell proliferation and even increases splenocyte proliferation in vitro [9], [35], [42]. We show herein that specific calpain inhibition delays melanoma cell growth both in vitro and in vivo.

Surprisingly, whereas in murine experimental models of cancer, tumor size usually correlates to metastatic dissemination, we observed a discrepancy between the slow tumor growth and the high rate of metastatic dissemination in calpastatin transgenic tumors. We evidenced that calpain inhibition in melanoma cells (i) confers resistance to death when exposed to immune cells and (ii) increases paradoxically migration, both mechanisms potentially leading to locoregional metastasis. The first mechanism is consistent with the abundant literature focusing on calpain involvement in cell death by apoptosis or necrosis [10], [11]. The observation that calpain inhibition increases tumor cell migration is unexpected since most reports show that calpain inhibition is much more likely to reduce cellular migration, as we previously observed in epithelial cell lines and splenocytes [9], [35]. However, it has been shown in cells with high calpain activity, such as neutrophils, that calpain inhibition induces cellular adhesion and rapid chemokinesis [43]. Cellular migration requires a subtle balance between cellular focal adhesions and detachment from extracellular matrix. Calpain-mediated proteolysis of paxillin has been described to negatively regulate focal adhesion dynamics and cell migration [44]. One may hypothesize that tumor cells with high calpain activity have low focal adhesion dynamics since focal adhesion components are known targets for calpains. A limitation of calpain activity would therefore reinforce migration properties and increase metastasis towards regional lymph nodes.

Additional mechanisms might explain how calpastatin expression increases metastatic dissemination. For instance, we and others have previously demonstrated that calpain inhibition increases the level of heat shock protein 90 (Hsp90) by limiting the proteolytic cleavage of ∼10 kDa C-terminal domain [45]. Since Hsp90 secretion, which requires a C-terminal motif, is essential for B16-F10 melanoma metastatic dissemination to regional lymph nodes, calpain inhibition would amplify this process [46]. This and other hypotheses all merit further investigation.

Beyond the properties of tumor cells themselves, the implication of calpains in host cells is of importance. We observed that specific calpain inhibition resulted, as expected, in a limitation of tumor angiogenesis, consistent with the observed limitation of tumor growth. We and others have recently studied some molecular mechanisms involving calpains in angiogenesis, especially in CalpTG mice [3], [8]–[9]. Calpains have been shown to be essential mediators of VEGF signalling in endothelial cells. Of notice, we also observed a decrease in immune cells infiltrating melanoma, which are essential to limit tumor extension and metastatic properties. We have previously shown that calpain inhibition by calpastatin transgene expression limits lymphocyte and NK cell migration toward inflammatory sites [35], [37]. Moreover, we have evidenced that calpain inhibition limits interleukin-17 (IL-17)-producing (Th17) cell polarization and promotes T regulatory cell induction, due to the inhibition of calpain-dependent degradation of the common cytokine receptor γ chain [35]. Since IL-17 and Th17 pathway seem to exert anti-tumor properties, it could be hypothesized that immune cells overexpressing calpastatin have reduced ability to limit tumor cell viability and metastatic dissemination. The antitumor effect of IL-17 is not direct, consistent with our in vitro experiments that did not demonstrate any functional deficiency in cytotoxic properties of immune cells with reduced calpain activity. Rather, the cytotoxic function of Th17 cells against melanoma is due to their ability to enhance antigen presentation by dendritic cells, leading to increased capture of tumor antigens, which are then presented to tumor-reactive CD8+ T cells in the draining lymph nodes [47]. These effector CD8+ T cells migrate back to the tumor where they exert a cytotoxic effect. Consistent with that, we observed a marked decrease in the number of CD8+ T cells infiltrating melanoma in CalpTG mice. The poor tumor infiltration by immune effector cells in CalpTG mice correlates with tumor escape from immune response and may therefore explain, at least partly, the higher metastatic dissemination rate in these mice.

Our findings thus point towards a process in which calpain inhibition in melanoma and host cells promotes both primary tumor growth inhibition and more invasive metastatic disease. Very interestingly, these results are reminiscent of the recent experimental evidence that VEGF-targeted drugs inhibit primary tumor growth but promote tumor invasiveness and metastasis [48]. They comply with the fact that calpain inhibition blunts VEGF signalling as well, and potentially explain the contradictory reports on the benefit of calpain inhibition in cancer models [9].

Therapeutic calpain inhibition comes of age soon in the field of cardiovascular and neurodegenerative diseases therapy [49]. We and others have previously shown that specific calpain inhibition protects against pathological arterial wall remodelling and there is now evidence that pharmacological calpain inhibition may improve cognitive functions in Alzheimer disease models [37], [49], [50]. Since calpains are overexpressed in many tumor tissues, synthetic conventional calpain inhibitors could have been considered as a promising therapeutic tool against cancer, as well [51]. Our results highlight some of the mechanisms by which the calpain/calpastatin system controls melanoma growth and metastatic dissemination. Clearly, calpain inhibition limits tumor growth and neo-angiogenesis, and could therefore prevent the initial growth of melanoma. These results are consistent with recent description of molecular mechanisms involving calpains in angiogenesis [3], [9]. By contrast, inhibition of calpains protects tumor cells against death and limits immune cells motility, thereby increasing melanoma cell dissemination. The role of calpains in apoptotic processes and cell death has been extensively studied but our observation that calpains, are essential for immune cells motility toward their targets, i.e. solid tumor, is original and corroborates our previous observations in allograft immune response [35]. Further studies will be necessary to address whether our experimental results apply to other solid tumors models or human cancers. Overall, our results evidence that studies of the calpain/calpastatin in cancer models should distinguish the role of calpains in tumoral cells and in host tissues/immune system. The development of new specific calpain inhibitors with potential medical applications should take into account this complexity.

## Materials and Methods

### Mice and induction of melanoma tumors

Studies were conducted in male 2-months old C57BL/6 mice. They were housed in a constant temperature room with a 12-h dark/light cycle and fed ad libitum on standard mouse chow. Calpastatin transgenic (CalpTG) mice were created in the laboratory using the cDNA clone of rabbit calpastatin inserted on the PCI expression vector, which includes a viral promoter (CMV immediate-early enhancer/promoter region) [36]. The presence and the expression of the transgene were identified in founder CalpTG mice by PCR and RT-PCR analysis, respectively [36]. All CalpTG mice used in these studies were backcrossed into the C57BL/6 background more than nine generations. CalptTG and WT were not littermates. To avoid genetic drift, CalpTG mice were therefore obtained from new frozen embryos within months before the study, sharing the same genetic C57BL/6 background than C57BL/6 control mice and bred in similar conditions.

One million tumoral B16-F10 cells (ATCC, USA) were injected subcutaneously under isoflurane anesthesia at the upper right part of the back of C57BL/6 control or CalpTG mice. These cells share the same C57BL/6 genetic background than mice. All procedures involving these animals were conducted in accordance with national guidelines and institutional policies. This study was carried out in strict accordance with the recommendations of the Institut National de la Santé et de la Recherche Médicale and the *in vivo* procedures were approved by the local ethical committee (CREEA Ile de France N°3). The sacrifice was performed under sodium pentobarbital anesthesia, and all efforts were made to minimize animals suffering. Tumor size was measured between days 9 and 16 by using a micrometer. Tumor volume was calculated from radius of the 3 axis: Volume =  4/3x(π)x(r1)x(r2)x(r3).The follow-up was performed until day 16. Tumors and lymph nodes were harvested at this time after sacrifice. Survival studies were performed along the same protocol. For ethical reasons and due to the tumor size, the survival analyzes were performed until day 30. Mice on the point of death and exhibiting signs of distress were sacrificed.

### Cell cultures and induction of stable transgenic cell lines

B16-F10 cells were cultured in RPMI medium (Gibco, France), containing 10% fetal bovine serum (Biowest, France) and supplemented with Hepes1M (Gibco,10 µl/ml) and penicillin/streptomycin (Gibco, 5 µl/ml).

Stable double tranfections were performed in B16-F10 cells with (i) 3 µg of PCI expression vector including a CMV promoter and the cDNA clone of rabbit calpastatin or empty control plasmids (PM 194, pCI-neoMammalian Expression Vector, Promega, UK) altogether with (ii) 0.3 µg of another plasmid including a neomycin resistance gene cassette. Calpastatin cDNA was the same cDNA previously used to generate transgenic mice. Double transfections were performed using a Nanofectin Kit according to manufacturer's instructions (PAA Laboratories, Austria). Cells were cultured with geneticin (G418, Merck) for 3 weeks and clones transfected by Calpastatin cDNA or control plasmids (controls) were selected. The efficiency of the transfection was assessed by quantitative RT-PCR. All experiments with calpastatin-transfected B16-F10 cells were performed with at least 2 different transfected clones.

### Quantitative RT-PCR

Quantitative RT-PCR. RNA was extracted from B16F10 cells using RNeasy Micro Kit columns (QIAGEN, Hilden, Germany). By using a reverse transcriptase (Fermentas, Saint Léon-Rot), cDNA was obtained from RNA and then amplified in a thermocycler (LightCycler 480, Roche Diagnostics) as follows: 95°C for 5 min followed by 45 cycles at 95°C for 15 s and 60°C for15 s, 72°C for 15 s, 96°C for 5 s and 60°C for 1 min, by using SYBR Green (Roche Diagnostics) and specific primers for mouse *Capn1*:F:AGTGGAAAGGACCCTGGAGT and R: TCTCGTTCATAGGGGTCCAC, mouse *Capn2* F: TGGCTTCGGCATCTATGAG and R: AAGTTTTTGCCGAGGTGGAT, mouse *Capn3 var a/b/c* F: TTGTGAGAATCCCCGGTTTA and R: TGCAAGAAACCAGCAGTCC, mouse *Capn4 or Capns1* F: GGTTTTGGCATTGACACTTG and R: TTGCCTGTGGTGTCGCTAT, mouse *Capn5* F: CGCACTGTGCTCTGCATC and R: AAGAAGGGGAGGCACCTG, mouse *Capn6* F: GCATTTTCCTGTTTGGCTGT and R: TGATCCTTGTGGTTGGGAAT, mouse *CapnN7* F: ACAAGACTGATGGCAAGAAGG and R: GTCAAGTAATGAGGGCTGTTAATTC, mouse *Capn8* F: ATAGGCTATGCTGTCTACCAGATTC and R: CCAGGTGCTCATCGGTGT, mouse *Capn9* F: GGACCGACATTTGCCAAG and R: GAGGGTTAGGGAGGCAATG, mouse *Capn10* F: TGTCTAATCAGCTGCTCTGTGC and R: ATGAAGGCATGGAACTCTCC, mouse *Capn11* F: TCTAGGTGTTCATCTGATAGATAGCC and R: TCCTTTTTCAGCCCAAGAGA, mouse *Capn12* F: AAAGGGGTGGAATGGAAGAG and R: GGCACACATCTGTTCTGCTC, mouse *Capn13* F: AACCTGGTCATGTACAGCTGAG and R: CGAGTGACCACTGGGAACA, mouse *Cast* F: TCGCAAGTTGGTGGTACAAG and R: CTCCCCAAACTTGCTGCTT, rabbit Cast F: AGCCAGCAAGTCGCTCAG and R: CCATCTCTTTGCTGATTGGAA. *Gapdh, Gusb* and *Rpl32* (respectively F: AGCTTGTCATCAACGGGAAG and R: TTTGATGTTAGTGGGGTCTCG, F: CTCTGGTGGCCTTACCTGAT and R: CAGTTGTTGTCACCTTCACCTC and F: GCTGCCATCTGTTTTACGG and R: TGACTGGTGCCTGATGAACT) were the housekeeping genes used for normalization. Results shown are expressed as 2-ΔΔCt and *Gapdh* was the housekeeping gene used for normalization.

### Western Blots

For western blot analysis, cell lysates were prepared by scraping cells into an ice-cold protease inhibitory buffer. Protein quantification was performed according to the standard Bradford technique. Twenty five micrograms of proteins were separated by electrophoresis on Novex BisTris 4–12% gels (NuPAGE, Invitrogen, San Diego, CA, USA) as described by the manufacturer and transferred onto a PVDF membrane (Immobilon-P, Millipore, Billerica, MA, USA) prior to detection of the following proteins with a specific primary Ab: Spectrin (Chemicon international; 1∶1500), Actin (Imgenex; 1∶2000), Calpastatin (Affinity Bioreagents; 1∶200), and peroxidase labelled anti-IgG secondary Ab (Amersham, dilution 1/4000 and GE Healthcare 1/4000). Thereafter, the membrane was developed with the ECL plus detection reagent (Amersham Biosciences, Piscataway, NJ, USA).

### Immunohistochemical analyzes

Melanoma tumors, local and regional lymph nodes were snap frozen and cut into 3 µm sections. Axillary lymph nodes histology was analyzed to determine their metastatic status. Endothelial cells were immunostained with purified rat anti-mouse CD31 antibody (MEC 13.3; BD Biosciences), rabbit anti-CD3 (1/200, Dako), rat anti-CD68 (1/500, Abcam,USA), rat anti-NK cells (1/500 BD Biosciences, USA), rat anti-CD4 (1/500 BD Pharmigen, USA) and rat anti-CD8 (1/600, Serotec, USA). Samples were revealed with Single Stain Mouse MAX PO (rat, goat or rabbit) Histofine (Nichirei Biosciences). Density of tumor vessels and immune cell counts were determined on pictures at 200×magnification by using Image J software. A grid was applied to blinded pictures and two independent observers trained in pathology performed cell and vessel count in 10 fields/mouse. The mean value of the ten fields was considered as a unique variable. The mean number of cells/HPF (n = 10 experiments) is shown.

### Calpain activity assay

Calpain activity in B16-F10 cells was measured as previously described, i.e. by both measuring the calpain-specific cleavage of fluorescent AMC substrate and by measuring the accumulation of 145/150-kDa spectrin BDP by Western blot analysis [13], [37].

### Cell proliferation

To perform BrdU incorporation assays, B16-F10 transgenic and control cells (50×10^3^ cells/well) were cultured in medium. BrdU was added to the wells at the later time. After further incubation (24 hours), cells were fixed, denatured, and immunostained with the anti-BrdU antibody (Cell Proliferation ELISA, BrdU, Roche). DNA synthesis was quantified by photometry according to manufacturer's instructions.

### Apoptosis

Melanoma cells were exposed to 50 µg/mL mitomycin C for 24 hours. Apoptotic cells were quantified by flow cytometry by using the Annexin V-FITC Apoptosis Detection Kit (Sigma, USA) according to manufacturer's instructions, FACScalibur flow cytometer and CellQuest Software (BD Biosciences).

### Cell migration

Melanoma monolayer repair assay: 7×10^4^ B16-F10 transgenic and control cells were cultured to confluence in each of the two wells of a Culture- Insert (Ibidi). After 24 h, the Culture Insert was removed and the cell monolayer including a central cell-free gap of 0.5 mm was covered with fresh medium. Gap surface area was analyzed at 0, 2, 4, 6, 8 and 10 hours by phase contrast microscopy and Image J software® (NIH).

### In vitro cytotoxic T-cell response

B16-F10 transgenic and control cells, first labelled with 100 µCi Na^51^CrO_4_ (GE Healthcare) for 90 min at 37°C, were added (2×10^4^ target cells in 200 µL) to each microwell of splenocytes from C57BL/6 WT or CalpTG mice previously immunized against one million B16-F10 control melanoma cells (Ctrl-Mel) injected 10 days before (4 ×10^5^ effector cells in 200 µL), allowing an effector/target ratio of 20∶1. After a 5 h incubation period at 37°C, the plates were centrifuged and ^51^Cr was detected in supernatants and cells by gamma count (LKB 1282 Compugamma CS). Results are expressed as% specific lysis, i.e. 100×(sample–spontaneous)/(maximum–spontaneous)^51^Cr release.

### Statistical analysis

Data are expressed as mean ± SEM. Means and SEM were generated from independent experiments. RT-PCR data were analyzed by the non-parametric Kruskal–Wallis and Mann–Whitney test. Other results were analyzed by Mann-Whitney or student bilateral t test and ANOVA when distribution was normal, and Fisher's exact test for comparison of proportions. Survival was analyzed by log-rank tests. Results with P<0.05 were considered statistically significant (Statview and graphpad Prism softwares).
